# Effects of iodinated copper-based foliar fertilizers on iodine content, yield and quality of lettuce (*Lactuca sativa* L.)

**DOI:** 10.3389/fpls.2026.1783391

**Published:** 2026-03-18

**Authors:** Anmeng Liu, Guobo Sun, Guofeng Ma, Min Zhang, Zhiguang Liu, Lingyun Tang

**Affiliations:** 1 Shandong Agricultural University, Taian, China; 2 Institute of Agricultural Resources and Regional Planning, Chinese Academy of Agricultural Sciences, Beijing, China; 3 Taishan Polytechnic, Taian, China; 4 Pingdu Branch of Qingdao Ecological Environment Bureau, Qingdao, Shandong, China

**Keywords:** biological effect, copper-based compounds, foliar fertilizer, iodine, lettuce, quality, trace element

## Abstract

Milk, dairy products, and certain marine foods are important dietary sources of iodine for humans. However, iodine intake is strongly influenced by dietary habits, local food availability, and iodine fortification policies, and ensuring adequate iodine intake remains a challenge in populations with low consumption of dairy products or seafood or in regions where salt iodization is limited. Under these circumstances, iodine-biofortified vegetables may serve as an important dietary source of iodine, and fertilization is an effective strategy to enhance their iodine content. In this study, using lettuce as a test crop, we conducted experiments over two growing seasons to evaluate an improved iodinated copper-based foliar fertilizer derived from traditional Bordeaux mixture (BDM), with treatments differing in iodine form and concentration. The results showed that the iodinated copper-based foliar fertilizers reduced copper concentrations in shoots and roots by 57.6%–91.57% and 61.56%–91.24%, respectively, compared with BDM, thereby reducing copper accumulation in plants and the potential risk of soil contamination. Compared with deionized water (CK), iodine application with copper-based fertilizers significantly increased iodine levels in lettuce tissues. Application of 1 g/L and 2 g/L KI copper-based foliar fertilizer increased shoot iodine content by 99.91% and 191.95%, respectively, and root iodine content by 180.66% and 211.41%, respectively. In another growing season, shoot iodine content increased by 24.71- to 66.20-fold, while root iodine content increased by 1.52- to 2.50-fold. In terms of quality, 1 g/L KI copper-based foliar fertilization increased vitamin C content by 33.19%, whereas 2 g/L reduced nitrate content by 39.08%; soluble sugar content increased by 34.41% and 39.27%. However, high iodine concentrations exerted a suppressive effect on yield, with high-concentration iodine treatments consistently reducing yield by approximately 13.85%–35.84% relative to CK across the two growing seasons. Overall, iodinated copper-based foliar fertilization effectively enhanced iodine enrichment in lettuce while simultaneously reducing copper accumulation, suggesting its potential as a safer alternative to traditional BDM for iodine biofortification. Among the tested treatments, 2 g/L KI copper-based foliar fertilization produced the greatest improvements in iodine enrichment and quality traits, although this was accompanied by a reduction in yield, indicating that the trade-off between nutritional enhancement and yield penalty should be carefully considered when determining the optimal application rate.

## 1 Introduction

Leaves are recognized as key organs for nutrient absorption and redistribution in plants. Foliar fertilization provides a rapid and efficient nutrient uptake pathway. It is particularly suitable for micronutrients easily fixed or leached in soil and has become a widely used technique in modern agriculture (Hu et al., 2023). Copper (Cu) is an essential metal for humans, animals, and plants, although it is also potentially toxic above supra-optimal levels (Shabbir et al., 2020). Copper compounds such as the Bordeaux mixture have been used for over a century as one of the most widely applied copper fungicides in agriculture, particularly for controlling fungal diseases in vines, fruits, and other crops (Deliopoulos et al., 2010; Vogelweith and Thiéry, 2018). However, long-term, repeated, and excessive application of copper fungicides has led to excessive accumulation of copper in plants and soil, which may reach toxic levels for plants and humans.

Based on this background, an improved copper-based foliar fertilizer (CBFF) was developed by the National Engineering Laboratory for Efficient Utilization of Soil and Fertilizer Resources at Shandong Agricultural University. The CBFF is an improved Bordeaux-type formulation that, with the addition of adjuvants, dispersants, and pH buffers, enhances suspension stability and leaf adhesion, overcoming the traditional mixture’s cumbersome preparation, sedimentation, and phytotoxicity issues (Zhu et al., 2012). When applied to leaves, it is quickly absorbed and translocated, enhancing photosynthesis and protective enzymes while limiting copper buildup in soil (Yao et al., 2014). Fungal and bacterial diseases are controlled by its broad antimicrobial activity, and the impacts on soil enzymes are reduced while the soil ecosystem is improved through controlled copper release and optimized formulation (Ma et al., 2019). At present, favorable application results have been achieved with the above CBFF and its improved derivative formulations in crops such as radish (*Raphanus sativus* L.) (Tang et al., 2017), pepper (*Capsicum annuum* L.), cotton (*Gossypium hirsutum* L.) (Qiang et al., 2018), and peanut (*Arachis hypogaea* L.), leading to the grant of several related patents (Zhang et al., 2013; Zhang et al., 2012; Lu et al., 2015).

Iodine is an essential trace element for both humans and animals, as it is involved in the synthesis and metabolism of thyroid hormones. Both deficiency and excess intake can be harmful and may lead to thyroid diseases (Guo et al., 2025). Mild iodine deficiency can result in goiter and benign thyroid nodules, while severe deficiency may cause hypothyroidism, intellectual impairment, and reduced fertility (Hatch-McChesney and Lieberman, 2022). Conversely, chronic excessive iodine intake also has adverse outcomes, including the induction or aggravation of hypothyroidism (Li et al., 2025) and increased risks of hyperthyroidism (Sohn et al., 2024), papillary thyroid carcinoma, and papillary thyroid microcarcinoma (Kim et al., 2021). Public nutrition guidelines recommend that dietary iodine be obtained mainly from food sources. Adult iodine intake is generally recommended at 100–150 μg day^-1^, whereas intakes above approximately 600 μg day^-1^ may increase the risk of adverse effects (EFSA NDA Panel on Dietetic Products, Nutrition and Allergies (NDA), 2014). Maintaining intake within this range helps to prevent both deficiency and excessive exposure.

Currently, universal salt iodization is implemented in most countries and regions as the main strategy to prevent iodine deficiency disorders. In China, the introduction of iodized salt has effectively controlled iodine deficiency at the national level and contributed to a marked reduction in iodine-related health risks (Wu et al., 2025). However, excessive accumulation of inorganic iodine in food may lead to adverse health effects, while it becomes nutritionally beneficial when converted into biologically active organic forms. Studies have shown that consuming more than 5 g of iodized salt per day may increase the risk of thyroid nodules and even thyroid cancer (Wang et al., 2021). The World Health Organization recommends limiting daily salt intake to below 5 g (World Health Organization, 2022). Under this recommendation, relying solely on iodized salt presents certain limitations. Exploration of alternative and balanced approaches to iodine supplementation remains necessary.

In recent years, agricultural approaches aimed at increasing iodine concentration in food crops have attracted growing attention. Iodine losses during high-temperature cooking are lower in iodine-enriched vegetables than in iodized salt, and iodine derived from vegetables shows higher absorption and utilization efficiency in the human body (Li et al., 2018). Therefore, consuming iodine-enriched vegetables may serve as an effective strategy for improving iodine intake and preventing deficiency disorders from a nutritional perspective. Under soil cultivation conditions, exogenous iodine application increases iodine concentration in crops (Faridullah et al., 2023). Plant uptake and translocation efficiency, however, remain limited, and iodine transport from roots to edible tissues is relatively low (Dobosy et al., 2024). Foliar application of liquid iodine fertilizers results in higher iodine accumulation in the leaves of leafy vegetables compared with soil application (Tallarita et al., 2025) Iodine enrichment capacity in plants is not unlimited; high external iodine concentrations can reduce iodine accumulation, suggesting inhibitory effects at elevated doses (Zhang et al., 2023). Iodine uptake and distribution also vary according to the chemical form applied. Some studies indicate that IO_3_^-^ is absorbed more efficiently than I^-^ under low-iodine conditions (Dobosy et al., 2024). The choice of iodine source and application method therefore influences biofortification efficiency. Potassium iodide (KI) has been recommended at specific growth stages to produce iodine-enriched vegetables (Lawson et al., 2016), whereas IO_3_^-^ treatments have resulted in higher iodine concentrations in edible tissues of certain crops (Duborská et al., 2024).

Application of iodine fertilizers at suitable levels increases iodine concentration in crops and may contribute to improvements in crop quality. For example, in carrot, tomato, and pepper, iodine treatments at reasonable concentrations have been shown to raise vitamin C (Vc) and soluble sugar content (Roosta et al., 2025), enhance antioxidant capacity (Duborská et al., 2024), and reduce nitrate levels (Li et al., 2017a), thereby improving overall quality. Given that many vegetables exhibit pronounced iodine enrichment capacity, the development of iodine-enriched vegetables holds broad prospects; however, the specific mechanisms of the effects of iodine uptake and enrichment in plants require further study.

Copper-based formulations are widely used in agriculture as protective fungicides. Under humid and mildly acidic conditions, small amounts of dissolved Cu²^+^ may be released from copper deposits (Lamichhane et al., 2018). The released Cu²^+^ may react with available inorganic iodide (I^-^) present in atmospheric deposition or rainwater (Gilfedder et al., 2007), leading to the formation of poorly soluble cuprous iodide (CuI). This process could reduce the proportion of iodine remaining in bioavailable forms for plant uptake. The iodinated CBFF used in this study consists primarily of copper hydroxide (60%) and iodine-containing compounds (32%). Copper is present mainly as sparingly soluble copper hydroxide (Cu(OH)_2_) colloids, and the suspension is maintained under alkaline conditions (pH 7–10.5), which limit the dissolution of Cu^2+^. In addition, the incorporation of dispersants, surfactants, wetting agents, and anti-flocculation additives enhances suspension stability and regulates ion availability within the system. Although copper-iodide interactions cannot be completely excluded, the physicochemical properties of the formulation help retain iodine in plant-available forms. Iodine, supplied as potassium iodide or potassium iodate, is incorporated into the copper-based foliar fertilizer to enable both disease protection and iodine biofortification. This approach seeks to improve crop nutritional value while reducing copper-related environmental risks.

To evaluate the effects of the iodinated CBFF on leafy vegetables, lettuce was selected as the test material because foliar-applied iodine is absorbed directly through leaf tissues, and lettuce is commonly consumed fresh, thereby minimizing iodine losses during cooking. Through plot experiments, the impacts of different types and concentrations of CBFF on yield, agronomic traits, quality, and trace element content were compared, thereby enabling the identification of appropriate application schemes and providing a theoretical basis for its wider adoption and the production of iodine-enriched agricultural products.

## 2 Materials and methods

### 2.1 Materials

The experiment was carried out from October 2015 to February 2016 and from March to June 2016 at the pilot test base of the National Engineering Technology Research Center for Slow and Controlled Release Fertilizers of Shandong Agricultural University in Tai’an City, Shandong Province, China (117°41’8”E; 36°9’46.52”N; altitude: 130.5 m). The area has a warm temperate continental semi-humid and semi-arid monsoon climate, with an annual average temperature of 13.0 °C and an annual average precipitation of 750 mm. Daily meteorological data during the experimental period are provided in the **Supplementary Files**. The tested lettuce variety was “American Fast-Growing, ” and its growth period was 45 days. Both growing seasons were conducted in a temperature-controlled greenhouse with a set-point temperature of 25 °C. The tested fertilizer was a self-prepared controlled-release blended fertilizer for vegetables. The soil texture is silt loam (US classification), with a pH of 7.81 (soil-to-water ratio as 2.5:1), organic matter content of 10.03 g/kg, total nitrogen content of 0.79 g/kg, available potassium content of 108.41 mg/kg, available phosphorus content of 21.33 mg/kg, available copper content of 3.33 mg/kg, and total iodine content of 1.02 mg/kg.

### 2.2 Methods

#### 2.2.1 Experimental design

Eight foliar fertilizer treatments were included in the experiment: CK (spraying with deionized water), BDM (spraying with the traditional Bordeaux mixture), CBFF1 (spraying with an ordinary copper-based foliar fertilizer at a concentration of 1 g·L^-1^), CBFF2 (spraying with an ordinary copper-based foliar fertilizer at a concentration of 2 g·L^-1^), CBFF-KI1 (spraying with a copper-based foliar fertilizer containing potassium iodide at 1 g·L^-1^), CBFF-KI2 (spraying with a copper-based foliar fertilizer containing potassium iodide at 2 g·L^-1^), CBFF-KIO1 (spraying with a copper-based foliar fertilizer containing potassium iodate at 1 g·L^-1^), and CBFF-KIO2 (spraying with a copper-based foliar fertilizer containing potassium iodate at 2 g·L^-1^). Seedlings were raised in trays and were transplanted to 1 m × 1 m plots after the development of 5–6 true leaves, with 20 plants per plot. The application rates of N-P_2_O_5_-K_2_O were 180-90–150 kg·ha^-1^, and three replicates were included for each treatment.

One week after transplanting the lettuce, the CBFF and other solutions were first applied, the second and third application was performed at 7–10 intervals. Two growing seasons were conducted, hereafter referred to as growing season I and growing season II. In the growing season I, lettuce was transplanted on December 30, 2015; foliar sprays were carried out on January 8, 18, and 28, 2016. Harvesting took place on February 28, and was delayed due to prolonged growth under low winter temperatures. In the growing season II, transplanting occurred on April 26, 2016, followed by foliar spraying on May 7, 16, and 25, and harvesting on June 18. The CBFF and other solutions were applied uniformly over both adaxial and abaxial leaf surfaces using a handheld sprayer, with spray volumes of 15, 30, and 100 mL per plot for the first, second, and third applications, respectively, until complete coverage without runoff. Identical irrigation, insect control, and weed control practices were conducted using local agronomic practices. All treatments were subjected to identical cultural management throughout the growing period.

#### 2.2.2 Determinations

The lettuce plants were pulled out whole and rinsed thoroughly with tap water and then deionized water, and the surface moisture was dried off with paper towels. The fresh weight was measured. The clean lettuce plants were cut at the root neck, and the weights of the above- and underground parts were measured separately. Some of the aboveground part was stored at 4 °C for the determination of indicators such as soluble sugar, protein, Vc, and nitrate. Parts of the above and underground samples were chopped and placed in a bag, placed in an oven at 105 °C for 30 minutes, dried at 80 °C to a constant weight, and ground and sieved for the determination of other indicators. The photosynthetic and fluorescence indicators were measured three times before spraying the CBFF and once again before harvest.

Soil basic physicochemical properties were determined following conventional methods (Wei, 1992). Fresh and dry weights of the aboveground and underground parts were determined by weighing method. Total nitrogen and total phosphorus were measured using the discrete chemical analyzer Smartchem-200. Total potassium was determined by flame spectrophotometry. Iodine content was analyzed using the ferric thiocyanate–nitrous acid catalytic kinetic method (Weng et al., 2008). Nitrate content was determined by ultraviolet spectrophotometry (Li et al., 2000). SPADvalues were recorded using a SPAD-5200 chlorophyll meter. Vc content was determined by the 2, 6-dichloroindophenol titration method (AOAC Official Method 967.21, 2005). Soluble sugar content was measured by anthrone colorimetry (Yemm and Willis, 1954). Nitrate content was determined by ultraviolet spectrophotometry. Soluble protein content was analyzed using the Coomassie Brilliant Blue G-250 method (Bradford, 1976). Photosynthetic performance was measured using a LI-6400XT portable photosynthesis system.

#### 2.2.3 Data statistics and analysis

The experimental data were organized and graphed using Microsoft Excel 2019. Analysis of variance was carried out using IBM SPSS Statistics 28.0 (IBM Corp., Armonk, NY, USA). Duncan’s multiple range test was applied to determine significant differences among treatment means at the p < 0.05 level.

## 3 Results

### 3.1 Effects of different treatments on iodine and copper content in lettuce

Copper concentrations in lettuce were significantly increased following spraying with BDM and CBFFs. Compared with the BDM treatment, copper concentrations in shoots and roots were significantly reduced by 57.6%–91.57% and 61.56%–91.24%, respectively, following CBFF application, with the greatest reduction being observed under the CBFF-KIO1 treatment.

Iodine concentrations in both shoots and roots were significantly increased by iodine-containing CBFFs compared with CK. In the CBFF-KI1, CBFF-KI2, and CBFF-KIO2 treatments, shoot iodine concentrations were increased by approximately 1.0–58.2-fold, 1.9–66.2-fold, and 1.3–25.3-fold, respectively, relative to CK. Although no significant difference was observed for CBFF-KIO1 in the growing season I, shoot iodine concentration was increased to 24.7-fold relative to CK in the growing season II. Root iodine concentrations were also markedly elevated in the CBFF-KI1, CBFF-KI2, CBFF-KIO1, and CBFF-KIO2 treatments, with respective increases of approximately 1.5–1.8-fold, 2.0–2.1-fold, 0.8–2.0-fold, and 0.3–2.5-fold compared with CK.

Two-way ANOVA revealed that Cu and I concentrations in both shoots and roots were significantly affected by treatment (P < 0.01). Root Cu concentration was significantly affected by growing season, whereas no significant effect was observed for shoot Cu or I concentrations. Significant interaction effects between growing season and treatment were observed for most parameters (**Table 1**).

**Table 1:** The trace element content of lettuce under different treatments (DW).

Growing season	Treatment	Copper (mg·kg-1)		Iodine (mg·kg-1)	
		Shoot	Root	Shoot	Root
(I)	CK	<LOD f	<LOD f	11.18 ± 1.43 de	65.01 ± 1.64 c
	BDM	137.95 ± 3.67 a	175.72 ± 6.97 a	0.2 ± 0.2 f	57.12 ± 0.43 c
	CBFF1	31.94 ± 3.51 d	53.29 ± 2.41 c	16.17 ± 1.96 bcde	47.7 ± 3.73 c
	CBFF2	58.49 ± 4.9 b	67.54 ± 4.33 b	7.3 ± 2.72 ef	49.01 ± 7.16 c
	CBFF-KI1	11.63 ± 3.28 e	46.52 ± 0 c	22.35 ± 0.54 abc	182.46 ± 13.31 a
	CBFF-KI2	48.43 ± 0.05 c	51 ± 1.42 c	32.64 ± 7.33 a	202.45 ± 4.9 a
	CBFF-KIO1	14.47 ± 0.47 e	15.39 ± 0.94 e	20.12 ± 0.03 bcd	115.13 ± 3.9 b
	CBFF-KIO2	13.34 ± 0.99 e	33.19 ± 0.52 d	25.21 ± 6.28 ab	94.88 ± 17.72 b
(II)	CK	<LOD c	2.65 ± 1.53 f	0.67 ± 0.36 c	52.78 ± 10.15 c
	BDM	200.45 ± 72.05 a	239.47 ± 9.45 a	1.36 ± 1.36 c	29.07 ± 5.76 c
	CBFF1	5.07 ± 1.04 bc	94.64 ± 2.93 c	1 ± 0.42 c	56.26 ± 10.7 c
	CBFF2	12.43 ± 6.52 bc	132.79 ± 3.98 b	6.78 ± 4.91 c	26.72 ± 7.52 c
	CBFF-KI1	71 ± 39.19 bc	38.96 ± 3.78 e	39.66 ± 3.95 a	132.75 ± 6.61 b
	CBFF-KI2	27.42 ± 14.22 bc	93.95 ± 5.19 c	45.02 ± 4.31 a	155.69 ± 20.08 ab
	CBFF-KIO1	80.31 ± 11.3 b	85.88 ± 19.27 cd	17.22 ± 1.7 b	156.95 ± 28.44 ab
	CBFF-KIO2	60.07 ± 1.88 bc	110.53 ± 15.24 bc	17.64 ± 3.09 b	184.93 ± 26.67 a
Two-way ANOVA					
Growing season(G)		ns	**	ns	ns
Treatments(T)		**	**	**	**
G×T		*	**	**	**

Values are expressed as means ± standard error (SE). Means in a column for the same fertilizer treatment and the same growing season followed by different lowercase letters indicate a significant difference at p < 0.05. * and ** indicate the significance level at p < 0.05 and p < 0.01. ns means not significant.

### 3.2 Effects of different treatments on the biomass and root-shoot ratio of lettuce

**Figure 1:**
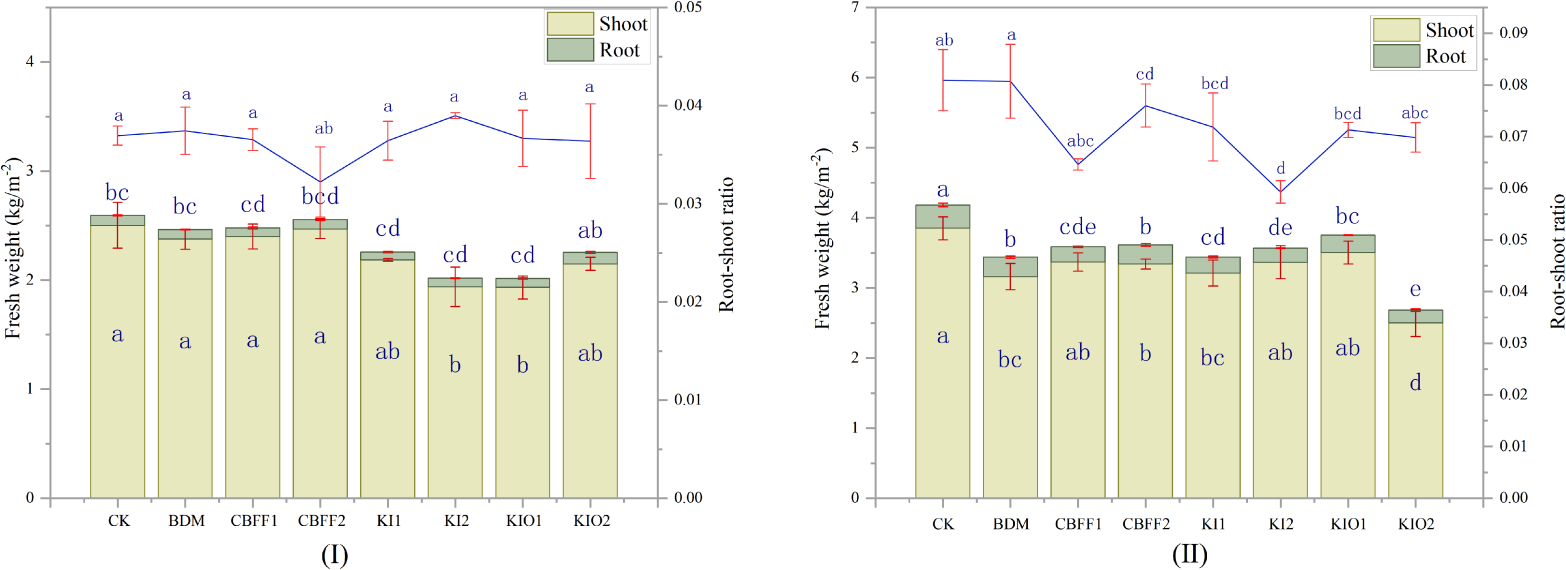
Effects of different foliar fertilizers on lettuce biomass and root–shoot ratio. Means with the same letter are not significantly different at the 5% level.

The total, aboveground, and underground fresh weights of lettuce were the highest in the CK treatment. In the growing season I, the total and aboveground fresh weights of lettuce under the CBFF-KI2 treatment were significantly reduced by 22.78% and 22.47%, respectively, while those under the CBFF-KIO1 treatment were significantly reduced by 22.46% and 22.73%. In the growing season II, the total fresh weights of lettuce treated with BDM and CBFFs (except CBFF-KIO1) were significantly reduced by 13.85%–35.84% compared with CK. The aboveground fresh weights of lettuce treated with CBFF1, CBFF-KI2, and CBFF-KIO1 showed no significant differences, whereas those treated with BDM, CBFF2, CBFF-KI1, and CBFF-KIO2 were significantly reduced by 17.95%, 13.28%, 16.63%, and 35.04%, respectively. The underground fresh weights of lettuce treated with BDM and CBFFs were significantly reduced by 15.66%–45.23% compared with the CK. The root–shoot ratio of lettuce treated with CBFFs (except CBFF1 and CBFF-KIO2) was significantly decreased by 5.04%–25.92% compared with the CK (**Figure 1**).

### 3.3 Effects of different treatments on the quality of lettuce in plots

In the growing season I, Vc content in the BDM, CBFF1, CBFF2, and CBFF-KI1 treatments was significantly increased by 53.17%, 18.52%, 58.48%, and 33.19%, respectively, compared with CK. In contrast, Vc content in the CBFF-KI2 treatment was significantly reduced by 20.32% compared with CK, whereas no significant differences were observed in the remaining treatments. Nitrate content in the BDM and CBFF-KIO2 treatments was significantly increased by 49.86% and 22.87%, respectively, compared with CK. Conversely, nitrate content in the CBFF1, CBFF-KI2, and CBFF-KIO1 treatments was significantly reduced by 33.30%, 39.08%, and 30.24%, respectively, compared with CK, whereas no significant differences were observed in the remaining treatments. No significant differences were observed in soluble sugar or soluble protein contents compared with CK across all treatments. However, in the growing season II, soluble sugar content in the CBFF-KI2 and CBFF-KIO2 treatments was significantly increased by 34.41% and 39.27%, respectively, compared with CK, whereas no significant differences were observed in the remaining treatments.

Two-way ANOVA showed that growing season significantly affected all quality parameters (P < 0.01). Treatment significantly influenced soluble sugars and Vc, while no significant main effect of treatment was observed for soluble protein and nitrate. Significant G×T interactions were detected for Vc and nitrate (**Table 2**).

**Table 2:** Quality indicators of lettuce under different treatments. (FW).

Growing season	Treatment	Soluble sugars content	Soluble protein	Vc	NO_3_^-^
		(%)	(mg·g^-1^)	(mg·100 g^-1)^	(mg·kg^-1^)
(I)	CK	1.33 ± 0.05 abc	8.89 ± 0.33 abc	38.44 ± 1.26 de	123.44 ± 13.99 cd
	BDM	1.27 ± 0.08 bc	9.61 ± 0.87 ab	58.88 ± 1.27 a	185.02 ± 3.61 a
	CBFF1	1.39 ± 0.44 abc	8.94 ± 0.91 abc	45.56 ± 0.41 c	82.33 ± 7.79 fg
	CBFF2	1.21 ± 0.04 c	8.37 ± 0.41 bc	60.92 ± 1.91 a	144.77 ± 11.75 bc
	CBFF-KI1	1.46 ± 0.29 abc	7.82 ± 0.79 bc	51.2 ± 1.69 b	104.34 ± 7.88 de
	CBFF-KI2	1.85 ± 0.16 a	9.39 ± 0.93 ab	30.63 ± 1.34 f	75.2 ± 2.63 g
	CBFF-KIO1	1.71 ± 0.22 abc	9.92 ± 0.67 ab	33.91 ± 2.12 ef	86.12 ± 0.3 ef
	CBFF-KIO2	1.83 ± 0.03 ab	11.01 ± 0.17 a	35.9 ± 1.09 def	151.67 ± 2.73 b
(II)	CK	2.47 ± 0.18 d	8.04 ± 1.05 a	27.91 ± 1.26 a	162.97 ± 29.22 ab
	BDM	2.55 ± 0.2 d	8.34 ± 1.29 a	30.57 ± 1.5 a	137.92 ± 27.17 ab
	CBFF1	2.74 ± 0.28 cd	5.75 ± 0.32 a	26.77 ± 0.66 a	82.22 ± 46.82 b
	CBFF2	2.96 ± 0.04 abcd	7.94 ± 0.98 a	27.25 ± 1.07 a	92.03 ± 61.86 ab
	CBFF-KI1	2.42 ± 0.09 d	6.84 ± 1.17 a	26.5 ± 1.06 a	138.96 ± 35.92 ab
	CBFF-KI2	3.32 ± 0.1 ab	6.6 ± 0.86 a	25.95 ± 2.18 a	208.19 ± 84.56 ab
	CBFF-KIO1	2.55 ± 0.17 d	7.7 ± 1.44 a	29.51 ± 1.57 a	267.09 ± 58.13 a
	CBFF-KIO2	3.44 ± 0.27 a	9.01 ± 1.94 a	26.12 ± 2.81 a	182.92 ± 54.28 ab
Two-way ANOVA					
Growing season(G)		**	**	**	**
Treatments(T)		**	ns	**	ns
G×T		ns	ns	**	*

Values are expressed as means ± standard error (SE). Means in a column for the same fertilizer treatment and the same growing season followed by different lowercase letters indicate a significant difference at p < 0.05. * and ** indicate the significance level at p < 0.05 and p < 0.01. ns means not significant.

### 3.4 Effects of different treatments on the nitrogen, phosphorus, and potassium contents of lettuce

No significant effects were observed on total nitrogen, total phosphorus, or total potassium contents in the aboveground parts of lettuce following spraying with BDM and CBFFs (except for the CBFF-KIO2 treatment). In the growing season I, total phosphorus contents in the underground parts under the CBFF1, CBFF-KIO1, and CBFF-KIO2 treatments were significantly increased by 48.53%, 35.86%, and 21.56%, respectively. Total potassium content in the underground parts under the CBFF-KIO1 and CBFF-KIO2 treatments was significantly increased by 26.92% and 30.06%, respectively, whereas no significant differences were observed in the remaining treatments.

**Figure 2:**
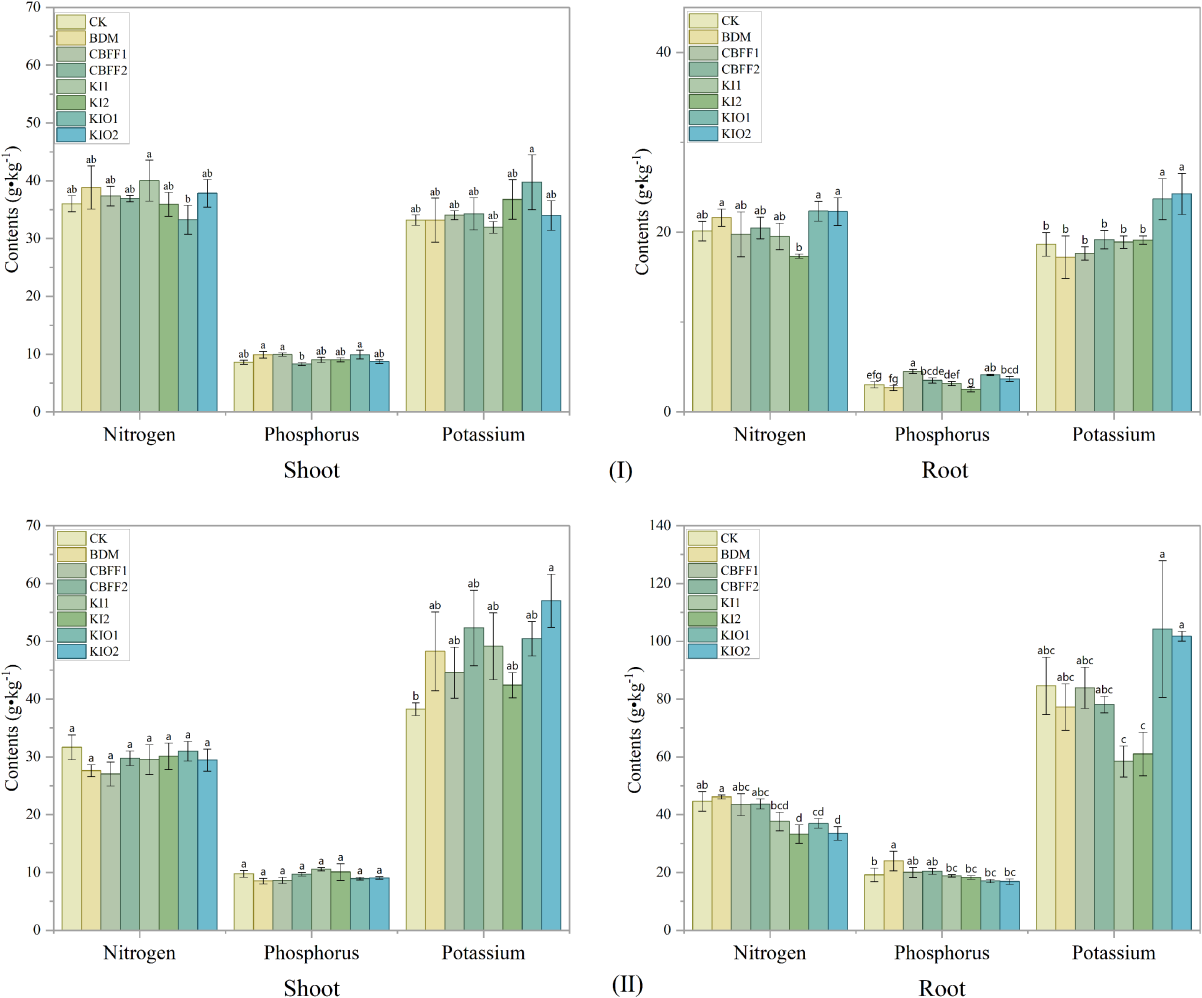
Total nitrogen, total phosphorus, and total potassium content in lettuce under different treatments. Means with the same letter are not significantly different at the 5% level.

In the growing season II, total nitrogen contents in the underground parts under the CBFF-KI2, CBFF-KIO1, and CBFF-KIO2 treatments were significantly reduced by 25.36%, 17.09%, and 24.89%, respectively, compared with CK. Total phosphorus content in the underground parts under the BDM treatment was significantly increased by 25.13% compared with CK. No significant effect was observed on total potassium content in the underground parts following spraying with BDM and CBFFs (**Figure 2**).

### 3.5 Effects of different treatments on the content of trace elements in the soil

**Figure 3:**
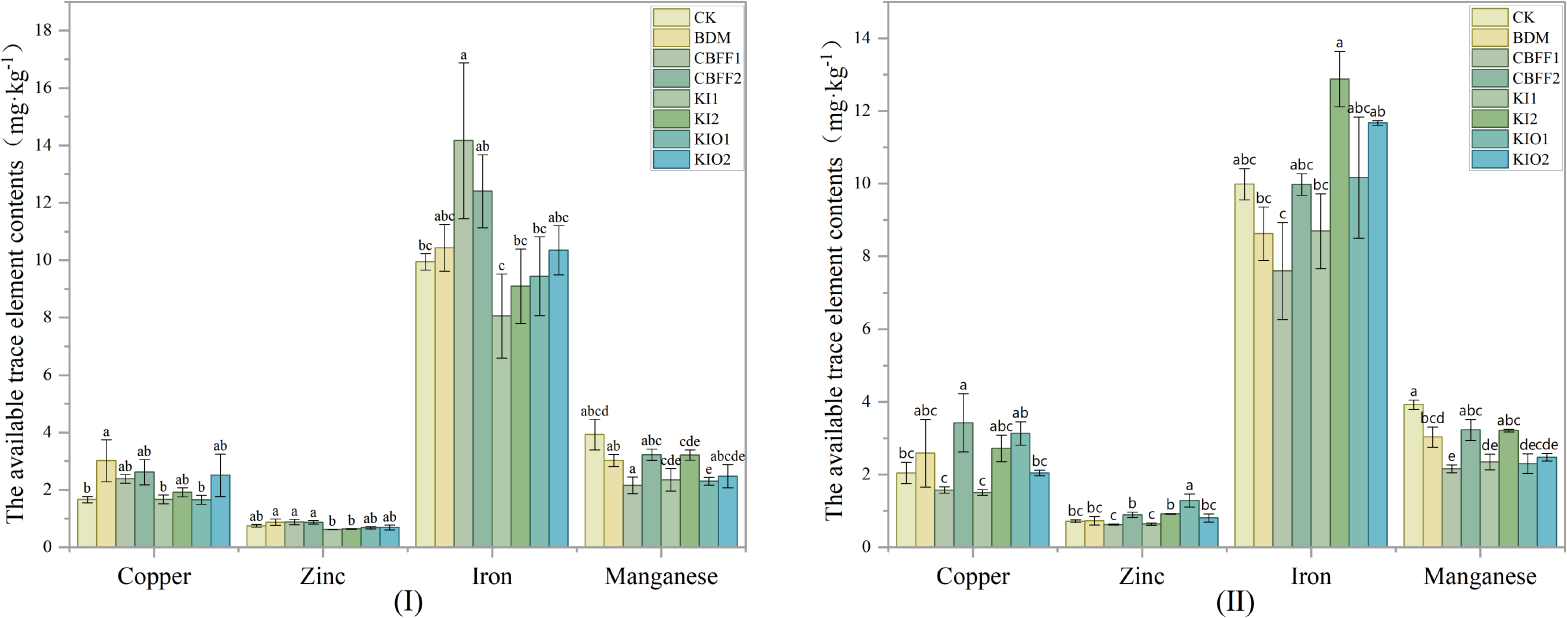
The available trace element content of soil under different treatments. Means with the same letter are not significantly different at the 5% level.

Spraying with BDM and CBFFs had an impact on the contents of trace elements in the soil. In the growing season I, the available copper content in the soil under the BDM treatment was significantly increased by 81.65% compared with the CK treatment, the available iron content in the soil under the CBFF treatment was significantly increased by 42.31% compared with the CK, and the available manganese content in the soil under the CBFF-KIO1 treatment was significantly decreased by 29.04% compared with the CK. There were no significant differences in the content of copper, zinc, iron, and manganese in the soil for the other treatments. In the growing season II, the available copper content in the soil under the CBFF2 treatment was significantly increased by 67.01% compared with the CK, and the available zinc content in the soil under the CBFF-KIO1 treatment was significantly increased by 80.96% compared with the CK. There were no significant differences in the available copper, zinc, and iron content in the soil for the other treatments. In the growing season II, the available manganese content in the soil decreased by 16.72%–44.92% compared with the CK, with the CBFF treatment leading to the greatest reduction. (**Figure 3**).

### 3.6 Effects of different treatments on the photosynthetic performance of lettuce

The photosynthetic performance of lettuce was not measured during the growing season I. Therefore, SPAD values of lettuce were measured at different time points during the growing season II. Chlorophyll content was regarded as an indicator of leaf yellowing, and variations in SPAD values were consistent with changes in chlorophyll content.

**Figure 4:**
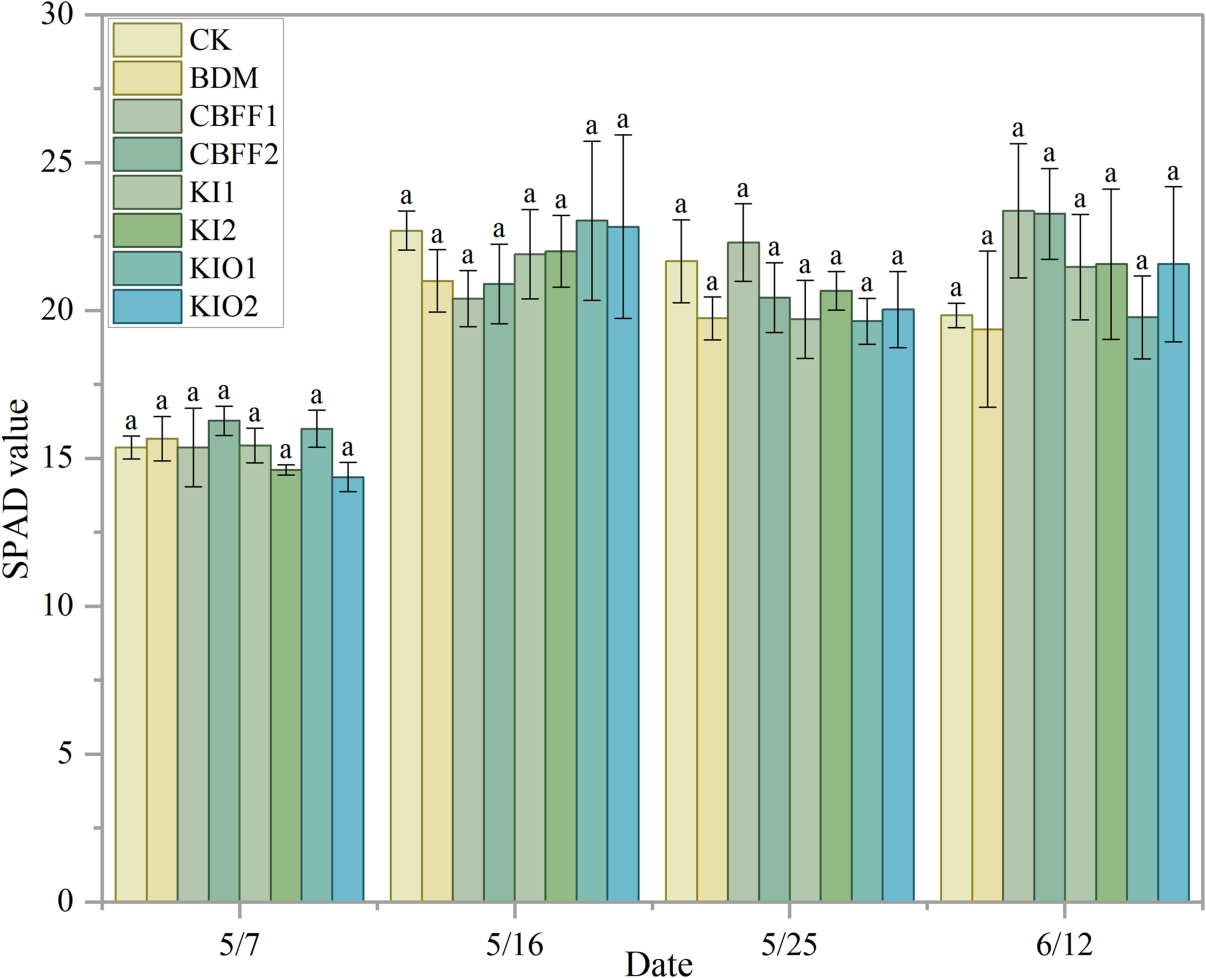
Changes in SPADvalue of lettuce under different treatments Means with the same letter are not significantly different at the 5% level.

During lettuce growth, SPAD values were first increased, then decreased, and finally tended to stabilize; however, no significant differences were observed among treatments. These results suggested that chlorophyll content of lettuce leaves was not significantly affected by CBFF treatments (**Figure 4**).

**Figure 5:**
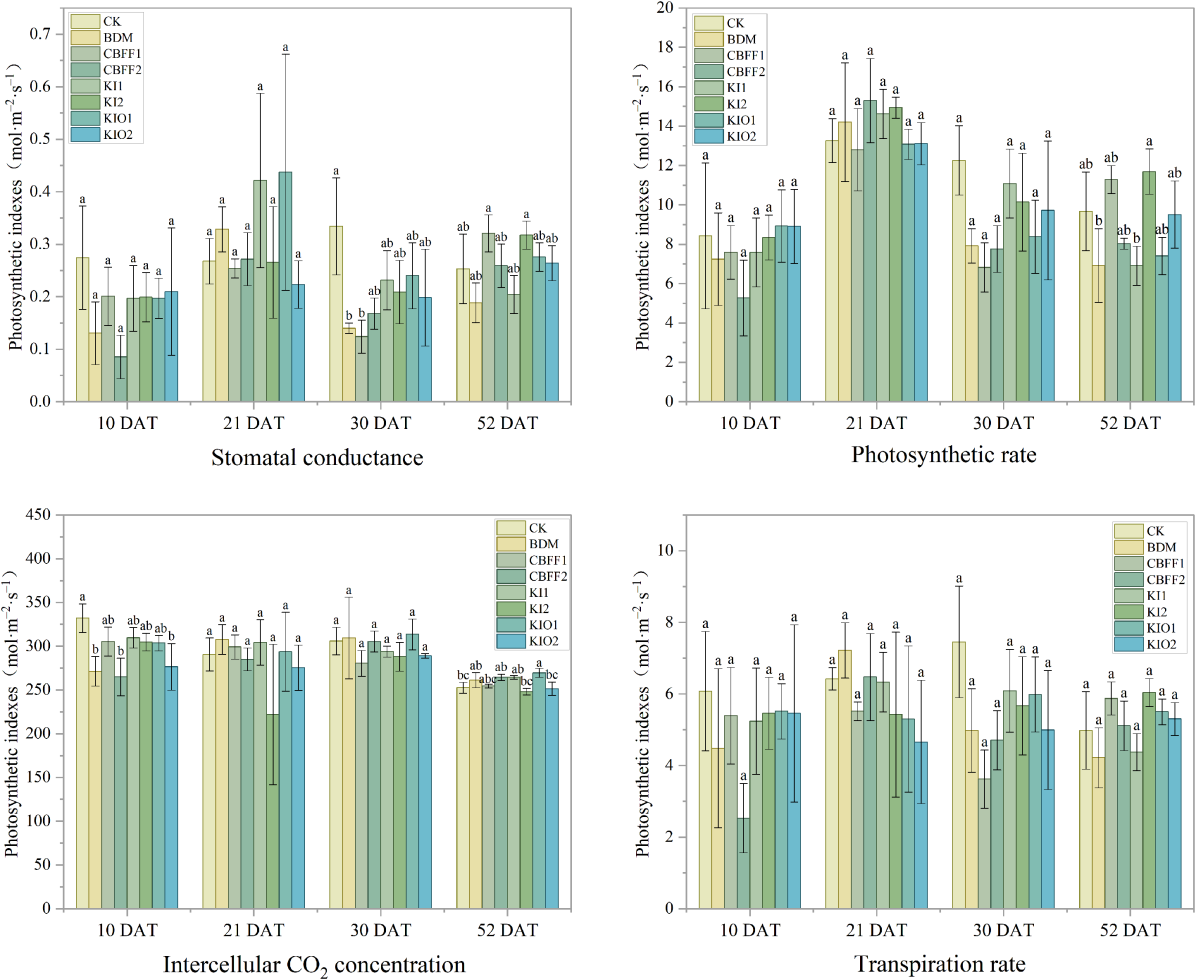
Changes in photosynthetic indexes of lettuce under different treatments. 1. Means with the same letter are not significantly different at the 5% level. 2. The test time refers to the number of days after transplanting the lettuce (DAT).

After transplantation, a brief lag phase associated with transplant shock is generally experienced by lettuce, followed by accelerated dry matter accumulation (Kerbiriou et al., 2013), and photosynthetic performance is considered to more accurately reflect growth status. No significant effects were observed on photosynthetic rate, stomatal conductance, or transpiration rate during the experimental period following spraying with BDM and CBFFs compared with CK. However, at the later growth stage (52 days after transplantation), intercellular CO_2_ concentrations were significantly increased by 3.41% and 6.73% under the BDM and CBFF-KIO1 treatments, respectively (**Figure 5**).

**Figure 6:**
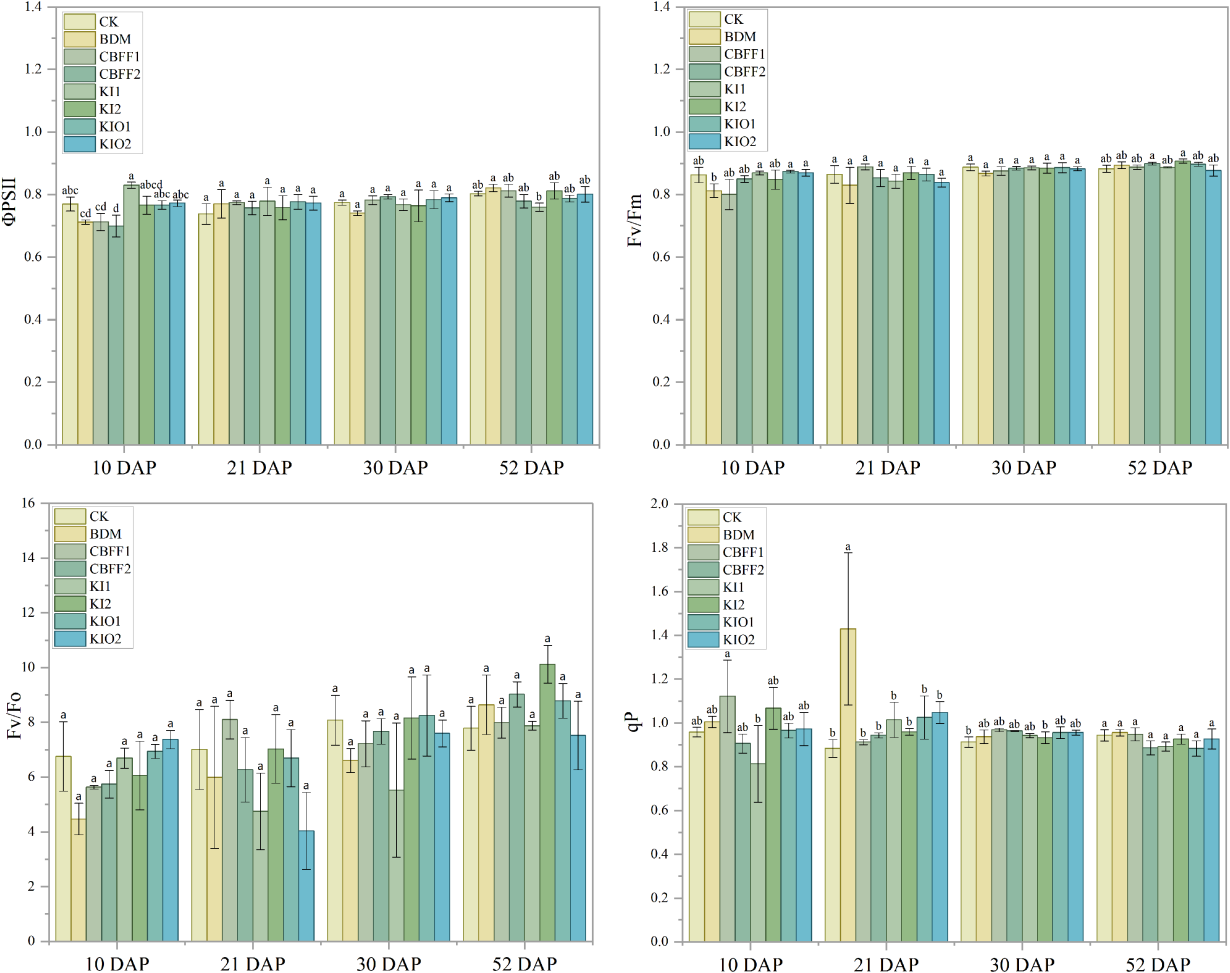
Changes in chlorophyll fluorescence parameters of lettuce under different treatments. 1. Means with the same letter are not significantly different at the 5% level. 2. The test time refers to the number of days after the lettuce was planted in soil (DAP).

Dynamic changes in photosynthetic physiological processes, including light absorption, transfer, dissipation, and energy distribution, were reflected by chlorophyll fluorescence characteristics. The changing trends of PSII quantum yield (ΦPSII), maximum photochemical efficiency (Fv/Fm), potential activity (Fv/Fo), and photochemical quenching coefficient (qP) were found to be consistent among treatments (**Figure 6**). On the 21st day, the qP value under the BDM treatment was significantly increased by 62.5% compared with CK; however, no significant differences were observed between other treatments and CK. On the 30th and 52nd days, no significant differences were observed among treatments. Overall, no significant effects were observed on photosynthetic and fluorescence characteristics following spraying with BDM and CBFFs (**Figure 6**).

## 4 Discussion

The main objective of this study was to investigate the effects of iodinated CBFFs on the yield, quality attributes, iodine and copper accumulation of lettuce. Data from two growing seasons were analyzed to assess their iodine biofortification efficiency and overall agronomic performance. It should be noted that both growing seasons were conducted in a temperature-controlled greenhouse with a set-point of 25 °C. It should be noted that both growing seasons were conducted in a temperature-controlled greenhouse with a set-point of 25 °C. However, actual greenhouse temperatures could deviate from the set-point due to outdoor conditions. In growing season I, owing to heat loss during winter, the greenhouse temperature was slightly lower than the set-point (approximately 21–24 °C during the daytime and 18–22 °C at night). In growing season II, the greenhouse temperature was slightly higher (approximately 25–28 °C during the daytime and 23–26 °C at night), which may have contributed to the seasonal differences observed.

The results from both growing seasons consistently demonstrated that the foliar application of iodinated CBFFs significantly enhanced iodine accumulation in lettuce tissues. Nevertheless, in the growing season I, iodine content in the aerial parts of lettuce was significantly reduced under the BDM treatment compared with CK. A plausible explanation for this finding is that lettuce leaves can directly absorb iodine from the air or from dissolved iodine in rainwater or saline solutions (Kiferle et al., 2021). Under humid or slightly acidic conditions, however, the BDM adhering to the leaf surface may release small amounts of Cu^2+^, which can react with I^-^ from the air or rainwater to form insoluble cuprous iodide (CuI), thereby decreasing the forms of iodine available for plant uptake (Lamichhane et al., 2018). In contrast, the CBFF-KI2 treatment resulted in the most pronounced iodine enrichment in lettuce, which may be attributed to the higher absorption rate of I^-^ compared with IO_3_^-^. Unlike the monatomic I^-^, IO_3_⁻ is a polyatomic anion with a more complex molecular structure, which may slow its diffusion from the cuticle to the epidermal cells (Wójcik and Wójcik, 2021). Furthermore, lettuce roots under the CBFF-KI2 treatment also showed the highest iodine content, primarily because I^-^ can be directly absorbed through anionic channels and chloride transporters energized by proton pumps, whereas IO_3_^-^ generally needs to be reduced to I^-^ in the roots before further utilization (Kato et al., 2013). These findings align with previous studies by Dobosy et al. on hydroponically grown cabbage (*Brassica oleracea* L.), who reported that under comparable conditions, I^-^ is more effective than IO_3_^-^ in increasing iodine concentrations in edible plant tissues (Dobosy et al., 2024).

In terms of yield, the effect of iodine on biomass largely depends on its application rate and the target crop (Medrano-Macías et al., 2016). For instance, in lettuce, Amaddin et al. reported that when the iodine concentration exceeded 7 mg L⁻¹, both fresh and dry weights decreased significantly, whereas at 5 mg L⁻¹ biomass increased markedly (Amaddin et al., 2024). Ortega-Ramirez et al. reported that foliar application of KI at 50 μM significantly increased biomass compared with the control, whereas 100 and 150 μM reduced total fresh biomass (Ortega-Ramirez et al., 2025). Similarly, Sánchez et al. found that 20 μmol L⁻¹ KI increased fresh weight by 77.7%, whereas 25 μmol L⁻¹ reduced biomass (Sánchez-Acosta et al., 2025). Vetési et al. reported that applying 0.5 mg L⁻¹ iodine to legumes suppressed fruit growth, although Fv/Fm was not significantly affected (Vetési et al., 2022). This observation aligns with our results, suggesting that iodinated CBFFs within the tested range did not adversely affect photosynthesis, and indicates that iodine biofortification under the present experimental conditions did not impair the core photosynthetic energy conversion system. Therefore, the observed yield reduction is unlikely to result from impaired photosynthetic capacity but may instead be associated with other physiological or environmental factors. Notably, intercellular CO_2_ concentration increased significantly under the CBFF-KIO1 treatment on day 53 after transplanting. This finding suggests that iodine application may exert a stage-specific regulatory effect during the later growth period by modulating gas exchange or carbon assimilation efficiency rather than directly inhibiting photosynthesis. However, the mechanisms underlying the iodine-induced changes in intercellular CO_2_ concentration remain unclear. One possible explanation lies in the multivalent properties of iodine. Because stomatal aperture is primarily regulated by K^+^ fluxes across guard cell membranes, I⁻ present on the leaf surface or within intercellular spaces may influence stomatal conductance and consequently intercellular CO_2_ concentration by altering membrane potential or competing with K^+^ for transport pathways (Lawson et al., 2016). Nevertheless, the precise molecular and physiological mechanisms require further investigation.

The quality of vegetables is largely determined by the chemical composition of their edible tissues, such as soluble solids, Vc, nitrates, and flavonoids (Gruda et al., 2025). In tomato, iodine supplementation increased soluble solid content and antioxidant capacity, which was associated with improved fruit quality (Ikram et al., 2024). Similarly, Smoleń et al. found that, in lettuce and other leafy vegetables, iodine treatments often produced combined effects, including increased iodine accumulation, elevated levels of Vc, soluble solids, and bioactive nutrients, as well as reduced nitrate concentrations (Smoleń et al., 2022; Dyląg et al., 2023). These findings are consistent with the present results, with iodine treatments showing differential effects on nitrate metabolism and antioxidant-related quality traits depending on concentration and formulation. The soluble sugar content was significantly increased under the CBFF-KI2 and CBFF-KIO2 treatments, while the Vc content was significantly higher under the CBFF2 and CBFF-KI1 treatments. However, during the growing season I, the Vc content under CBFF-KI2 was slightly lower than that of the CK. This may be because an appropriate foliar iodine application can enhance nitrate reductase (NR) activity, thereby promoting nitrogen assimilation and reducing nitrate accumulation in plant tissues (Patiño-Cruz et al., 2025). Concurrently, moderate iodine application also enhances the activity of L-galactono-1, 4-lactone dehydrogenase (GLDH) (Blasco et al., 2011), thus promoting the biosynthesis of VC. However, excessive iodine can hinder both Vc synthesis (Li et al., 2017b) and nitrate reduction metabolism (Blasco et al., 2010), ultimately leading to a decrease in Vc content and the accumulation of nitrite in lettuce.

Essential trace metals such as copper, zinc, iron, and manganese play critical roles in plant growth, stress resistance, photosynthesis, and various biosynthetic processes (Behtash et al., 2022). There are potential antagonistic interactions between copper and zinc or manganese, and zinc may also exhibit antagonism with iron (Shahriaripour and Tajabadipour, 2010). Therefore, monitoring the concentrations of Zn, Fe, and Mn was intended to assess the possible indirect effects of copper application on soil micronutrient balance and environmental safety. The observed decrease in Mn availability may reflect antagonistic interactions between Cu and Mn following repeated Cu inputs. In contrast, a significant increase in soil available Zn was observed only under the CBFF-KIO2 treatment, while no significant differences in Zn, Fe, or Mn were detected under the other iodine-containing treatments. Overall, these results suggest that the iodinated CBFFs did not cause widespread or systematic disturbances to soil micronutrient balance under the application rates used in this study.

Long-term and excessive use of copper-based formulations can cause substantial copper accumulation in soils, particularly in the topsoil within the root zone, thereby impairing soil quality, productivity, and ecosystem functions. However, in this study, the application of iodinated CBFFs resulted in no significant difference in available soil copper compared with the CK. This is because the formulations incorporated wetting agents composed of alkyl naphthalene sulfonates and anionic surfactants, and dispersing agents consisting of ammonium polyacrylate together with sulfonate-type and phosphate-ester-type anionic surfactants. Combined with particle-size reduction achieved through sand-mill grinding at a rotation speed of 1200 r·min^-1^ for 60 minutes, these modifications increased the specific surface area of the formulations. These physicochemical improvements reduced solution surface tension and leaf contact angle, thus enhancing droplet dispersion and foliar adhesion, which in turn minimized copper runoff and leaching into the soil (Yao et al., 2014). Across both growing seasons, iodinated CBFFs led to lower copper concentrations in both the shoots and roots of lettuce than the BDM.

In summary, iodinated CBFFs are effective in increasing iodine content and improving lettuce quality while simultaneously reducing the risk of copper toxicity. However, high concentrations pose a potential risk of yield reduction. Although slight yield declines were observed under certain high-concentration treatments, the production of iodine-enriched lettuce characterized by lower nitrate content and higher vitamin C levels may enhance product value and market differentiation. With growing consumer demand for functional and health-oriented agricultural products, such biofortified vegetables may command price premiums that compensate for moderate yield losses. Therefore, practical applications should balance iodine enrichment, product quality, yield stability, and soil ecological safety and determine appropriate formulations and application regimens accordingly. Future work should include cost–benefit analyses when yield gains are constrained, quantifying fertilization and spraying costs, quality-related price premiums, and per-area returns, alongside scenario analyses under different production contexts, to support the scaled and industrial application of iodinated CBFFs. Beyond lettuce, iodinated CBFFs may also hold application potential in other leafy vegetables, particularly those consumed fresh. Future research could further expand to different crop types and consumption scenarios, including both fresh consumption and processed products, in order to evaluate iodine stability, bioavailability, and quality performance under diverse post-harvest conditions, thereby enhancing the broader applicability and extensibility of this biofortification strategy.

## 5 Conclusion

Given the results from the two growing seasons, certain iodine-containing treatments demonstrated positive effects in increasing iodine concentration in lettuce, improving selected quality parameters, reducing nitrate content, and mitigating risks of copper phytotoxicity and soil contamination. Among all treatments, the application of 2 g L⁻¹ KI copper-based foliar fertilizer showed the most balanced performance in terms of yield, iodine biofortification efficiency, and nutritional quality. Overall, under the present experimental conditions, iodinated copper-based foliar fertilizers achieved efficient iodine biofortification while enhancing product quality and reducing copper accumulation compared with BDM. These findings highlight their potential as a practical foliar iodine supply strategy for lettuce and potentially other leafy vegetables.

## Data Availability

The raw data supporting the conclusions of this article will be made available by the authors, without undue reservation.
